# Asiatic acid protects against hepatic ischemia/reperfusion injury by inactivation of Kupffer cells via PPARγ/NLRP3 inflammasome signaling pathway

**DOI:** 10.18632/oncotarget.21151

**Published:** 2017-09-21

**Authors:** Ying Xu, Jun Yao, Chen Zou, Heng Zhang, Shouliang Zhang, Jun Liu, Gui Ma, Pengcheng Jiang, Wenbo Zhang

**Affiliations:** ^1^ Department of Laboratory Center, Affiliated People’s Hospital of Jiangsu University, Zhenjiang, China; ^2^ Department of Gastroenterology, Affiliated People’s Hospital of Jiangsu University, Zhenjiang, China; ^3^ Department of General Surgery, Affiliated People’s Hospital of Jiangsu University, Zhenjiang, China

**Keywords:** asiatic acid, NLRP3 inflammasome, PPARγ, hepatic I/R injury, Kupffer cell

## Abstract

Hepatic ischemia/reperfusion (I/R) contributes to major complications in clinical practice affecting perioperative morbidity and mortality. Recent evidence suggests the key role of nucleotide-binding oligomerization domain-like receptor (NLR) family pyrin domain-containing 3 (NLRP3) inflammaosme activation on the pathogenesis of I/R injury. Asiatic acid (AA) is a pentacyclic triterpene derivative presented with versatile activities, including antioxidant, anti-inflammation and hepatoprotective effects. This study was designed to determine whether AA had potential hepatoprotective benefits against hepatic I/R injury, as well as to unveil the underlying mechanisms involved in the putative effects. Mice subjected to warm hepatic I/R, and Kupffer cells (KCs) or RAW264.7 cells challenged with lipopolysaccharide (LPS)/H_2_O_2_, were pretreated with AA. Administration of AA significantly attenuated hepatic histopathological damage, global inflammatory level, apoptotic signaling level, as well as NLRP3 inflammasome activation. These effects were correlated with increased expression of peroxisome proliferator-activated receptor gamma (PPARγ). Conversely, pharmacological inhibition of PPARγ by GW9662 abolished the protective effects of AA on hepatic I/R injury and in turn aggravated NLRP3 inflammasome activation. Activation of NLRP3 inflammasome was most significant in nonparenchymal cells (NPCs). Depletion of KCs by gadolinium chloride (GdCl3) further attenuated the detrimental effects of GW9662 on hepatic I/R as well as NLRP3 activation. *In vitro*, AA concentration-dependently inhibited LPS/H_2_O_2_-induced NLRP3 inflammaosome activation in KCs and RAW264.7 cells. Either GW9662 or genetic knockdown of PPARγ abolished the AA-mediated inactivation of NLRP3 inflammasome. Mechanistically, AA attenuated I/R or LPS/H_2_O_2_-induced ROS production and phosphorylation level of JNK, p38 MAPK and IκBα but not ERK, a mechanism dependent on PPARγ. Finally, AA blocked the deleterious effects of LPS/H_2_O_2_-induced macrophage activation on hepatocyte viability *in vitro*, and improved survival in a lethal hepatic I/R injury model *in vivo*. Collectively, these data suggest that AA is effective in mitigating hepatic I/R injury through attenuation of KCs activation via PPARγ/NLRP3 inflammasome signaling pathway.

## INTRODUCTION

Liver ischemia/reperfusion (I/R) injury is a significant clinical problem that is frequently encountered during liver transplantation, liver resection, massive trauma, hemorrhagic shock, sepsis and cardiopulmonary failure. Typically, the pathology of I/R is characterized by a biphasic phase of inflammatory response [1, 2]. The initial phase occurs within 1-6 h after reperfusion, and is manifested by the release of various damage associated molecular patterns (DAMPs), activation of liver resident Kupffer cells (KCs) and their subsequent production and release of reactive oxygen species (ROS) as well as various mediators, such as cytokines, chemokines and adhesion molecules. This causes mild injury to the hepatic parenchyma, but the released soluble mediators result in the recruitment and activation of neutrophils and inflammatory monocytes, and also create a feedback loop that further activates hepatocellular stress kinase pathways, exacerbating injury [1, 2]. The current treatments for hepatic I/R at the bedside remain largely supportive and limited due to the complex mechanisms. To disrupt the inflammatory cascades at early stage of hepatic I/R seems to be a promising option. This is supported by evidence that inhibition of KCs or cytokine neutralization protected against hepatic I/R injury in animal studies [3-6].

Emerging evidence indicates that inflammasomes are important sensors and effectors in the pathology of ischemic liver [2, 7]. The prototypical inflammasome consists of a nucleotide-binding oligomerization domain-like receptor (NLR) molecule, apoptosis-associated speck-like protein containing a caspase recruitment domain (ASC) and pro-caspase-1. Assembled inflammasome is capable to activate caspase-1 and serves as a key platform for maturation of IL-1β and other cytokines [8]. Of those inflammasome-forming NLRs, NLR family pyrin domain-containing protein 3 (NLRP3) is best characterized by far. NLRP3 extensively involves in the recognition of numerous exogenous and host ligands, including ATP, DNA and uric acid crystals. The functional importance of NLRP3 in hepatic I/R has been supported by several lines of evidence. Firstly, interleukin (IL)-1 family cytokines (IL-1β and IL-18) acting downstream of inflammasome, are among the most important signal amplifiers due to their ability to induce secretion of other cytokines [9, 10]. Pharmacological blockade of either IL-1β or IL-18 has proved to be hepatoprotective against I/R injury [6, 11, 12]. In addition, IL-1β knockout mice exhibit significant reduction of I/R injury [13] and consistently, transfer of the IL-1 receptor antagonist gene into rat liver abrogates I/R injury [14]. Deficiency of key inflammasome components, such as NLRP3, ASC and pro-caspase-1 [6, 15, 16], or inhibition of active caspase-1 by YVAD [17], also protects mice against hepatic I/R injury. Thus NLRP3 inflammasome may serve as a novel therapeutical target for the treatment of hepatic I/R.

Asiatic acid (AA) is a pentacyclic triterpene derivative from the Chinese herb Centella asiatica. Centella asiatica and AA show a low risk of adverse side-effects and a long history of successful use both in traditional Chinese medicine and Indian Ayurvedic medicine. Previous studies have demonstrated that AA exhibits a variety of activities including antioxidant, anti-inflammation, neuroprotective and anticancer effect both *in vitro* and *in vivo* [18-22]. In particular, AA has been shown to protect against liver injuries in several animal models [23-28]. Emerging evidence demonstrates that AA inhibits NLRP3 inflammasome assembling and caspase-1 inhibition in macrophage [29]. In addition, AA has been reported to increase peroxisome proliferator-activated receptor (PPAR)-γ expression *in vitro* [30], a key regulator in energy metabolism and inflammation. Here, we set out to determine the role of AA on hepatic I/R injury, as well as to investigate the underlying mechanisms behind these putative effects.

## RESULTS

### Pretreatment of AA attenuates hepatic I/R injury and global inflammatory levels

**Figure 1:**
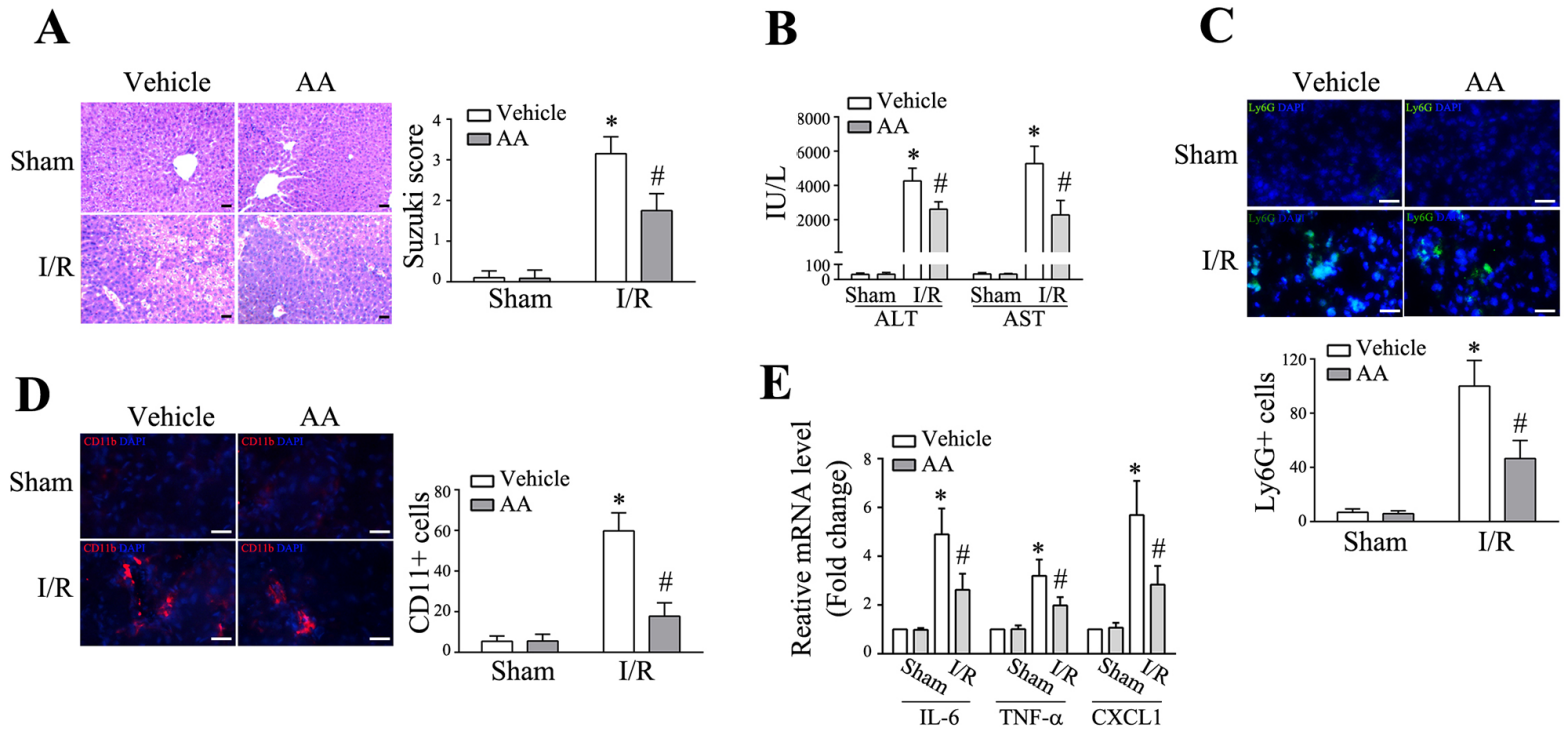
AA attenuates hepatic I/R injury and global inflammatory levels Male C57BL/6 mice were pretreated with either AA (30 mg/kg) or vehicle 1 h before hepatic I/R surgery. After 6 h reperfusion, liver tissues and serum samples were harvested. **(A)** Representative histological staining (H&E) of ischemic liver tissue. Results representative of 4-6 mice/group. Liver damage, evaluated by Suzuki’s histological score. Scale bar: 30μm. **(B)** Hepatocellular function in serum samples was evaluated by sALT and sAST levels (IU/L). Results expressed as mean±SD (n=4-6 samples/group). **(C)** & **(D)** Liver neutrophils and monocytes/macrophages were detected by immunofluorescent staining. Quantification of Ly6G+ cells and CD11b+ cells per HPF. Representative of 4-6 mice/group. Scale bar: 30μm. **(E)** RT-qPCR for detection of IL-6, TNF-α, and CXCL1 in ischemic livers (n=4-6 samples/group). * Significant difference (*P* < 0.05) compared with corresponding control. # Significant difference (*P* < 0.05) compared with I/R.

Previous studies have shown that AA exerts hepatoprotective effects in several animal models, which include CCl4-induced liver fibrosis, D-galactosamine/lipopolysaccharide (GalN/LPS)-induced hepatotoxicity, high-fat diet-induced hepatic steatosis, and Con A-induced hepatitis [23-26]. However, the effect of AA on hepatic I/R remains undefined. Thus, mice were challenged with a partial warm hepatic I/R insult. AA with a dose of 30 mg/kg body weight was chosen for the present study, according to previous observations as well as our preliminary experiments (Supplementary Figure 1). As shown in Figure 1A, I/R led to significant liver injury as assessed by histology in lobular edema, congestion, ballooning and hepatocellular necrosis 6 h after reperfusion. Certainly, Suzuki’s score as well as sALT and sAST levels (Figure 1B) were significantly increased. The baseline effect of AA was comparable to the corresponding controls. However, pretreatment of AA conferred significant histological improvement as well as reduction of sALT and sAST. Exaggerated inflammatory response, featured by increased expression of inflammatory mediators and infiltration of circulating leukocytes, is a hallmark of hepatic I/R injury. Indeed, I/R lead to markedly increased infiltration of Ly6G+ neutrophils (Figure 1C) and CD11b+ monocytes/macrophages (Figure 1D) compared to the corresponding controls. These effects could be greatly abolished by AA pretreatment. Consistent with these findings, AA pretreatment also abated I/R-induced increase in the mRNA levels of global inflammatory mediators, including IL-6, TNF-α and CXCL1 (Figure 1E). The reductions of these inflammatory cytokines and chemokines appeared to facilitate the improvement of the hepatic inflammation.

### AA decreases NLRP3 inflammasome activation in ischemic liver

**Figure 2:**
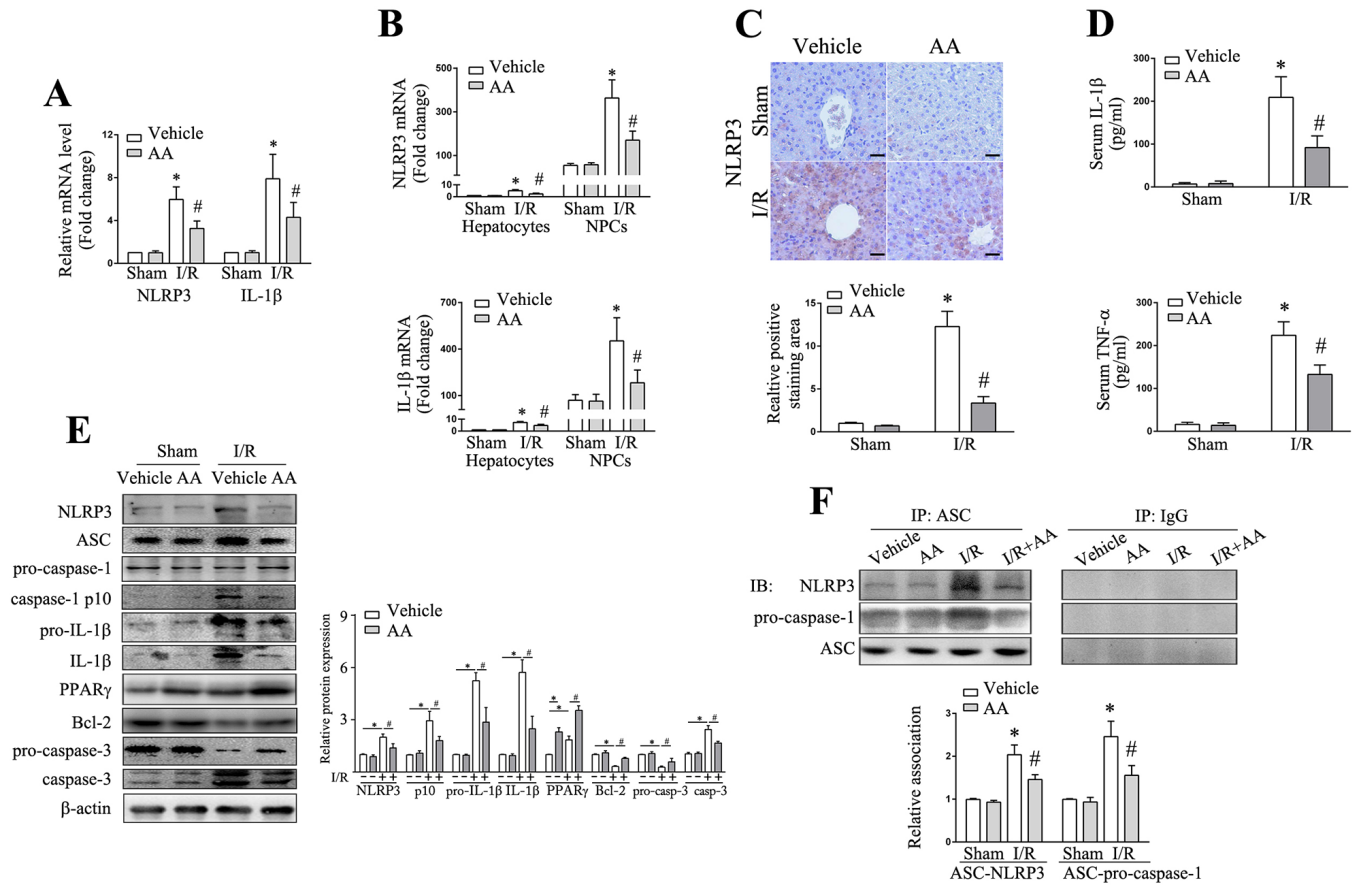
AA decreases NLRP3 inflammasome activation in the ischemic liver **(A)** Representative RT-qPCR for detection of NLRP3 and IL-1β in ischemic livers. **(B)** The hepatocytes and NPCs were isolated from ischemic livers after 6 h reperfusion. Relative NLRP3 and IL-1β mRNA expressions in each cell fraction were quantified. **(C)** Immunohistochemical staining of NLRP3 in ischemic livers. Results scored quantitatively by averaging area of positively stained cells (mean±SD)/field at ×200 magnification. Scale bar: 30μm. **(D)** ELISA analysis of IL-1β and TNF-α levels in animal serums. **(E)** Protein expressions of NLRP3, ASC, pro-caspase-1, cleaved caspase-1 p10, pro-IL-1β, IL-1β, PPARγ, Bcl-2, pro-caspase-3 and caspase-3 were detected using Western blot analysis. Relative density ratios of interested proteins were normalized to β-actin and then compared. **(F)** Liver lysates were immunoprecipitated using anti-ASC or control IgG antibody, and precipitated proteins were immunoblotted using anti-NLRP3 or anti-pro-caspase-1 antibodies. The signal intensities were quantified as the ratio to total amount of immunoprecipitated ASC. The results are presented as the mean±SD of 4-6 animals per group. Blots shown are representative of 3 experiments with similar results. * Significant difference (*P* < 0.05) compared with corresponding control. # Significant difference (*P* < 0.05) compared with I/R.

The NLRP3 inflammasome is considered to be a prominent and early mediator of inflammatory responses corresponding to tissue damage [2, 7]. I/R induced a wide range of DAMPs, many of which act to be competent to activate NLRP3. The upregulated expression of NLRP3 and pro-IL-1β is a prerequisite for activation of NLRP3 inflammasome pathway [8]. As shown in Figure 2A, I/R induced a significant increase in the mRNA levels of NLRP3 and pro-IL-1β compared to the corresponding controls. Increased NLRP3 protein levels were also observed in ischemic livers as shown by immunohistochemistry analysis (Figure 2C). This was also supported by Western blot analysis in which I/R lead to increased protein levels of NLRP3 and pro-IL-1β in ischemic livers compared to the controls (Figure 2E). NLRP3-ASC complex binding pro-caspase-1 predisposes pro-caspase-1 to be autocleaved into its active form p10 and p20. Although the expressions of inflammasome components pro-caspase-1 and ASC showed no significant changes after I/R, significant increase in cleaved capase-1 p10 and mature IL-1β was observed in ischemic livers (Figure 2E) compared to the corresponding controls. Elevated IL-1β was also observed in serum post I/R (Figure 2D). Consistently, the association of NLPR3-ASC-pro-caspase-1 was increased in I/R mice in comparison with the controls (Figure 2F). All the above effects could be abolished by AA pretreatment. Serum TNF-α is readily assessed as a general marker of inflammation and organ damage during hepatic I/R. We showed that I/R-induced increase in serum TNF-α level could also be attenuated by AA (Figure 2D). As apoptosis resulting from aggravated inflammatory response significantly contributes to cell death during I/R injury besides necrosis, we also examined Bcl-2 and active caspase-3 expression in ischemic livers. As shown in Figure 2E, I/R induced significant activation of apoptotic signaling pathway, as manifested by decreased Bcl-2 and elevated caspase-3 expression compared to the controls. Again, AA could also attenuate this effect.

### Inhibition of PPARγ abolishes the protective effects of AA against I/R-induced inflammatory injury and NLRP3 inflammasome activation

**Figure 3:**
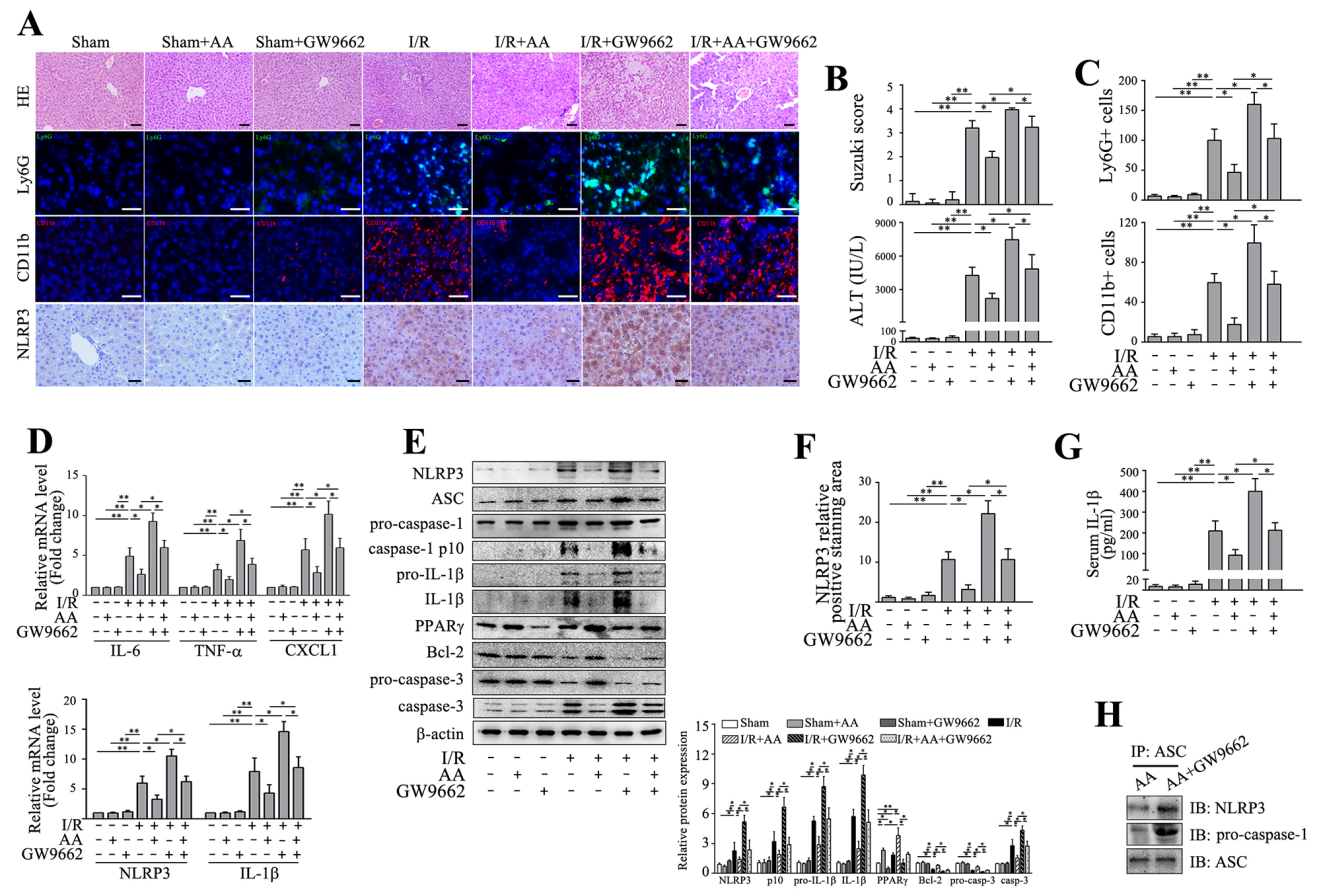
Inhibition of PPARγ abolishes the protective effects of AA against I/R-induced inflammatory injury and NLRP3 inflammasome activation Mice were pretreated with GW9662 (2 mg/kg, i.p.) or vehicle 30 min prior to treatment with AA, followed by an I/R insult. Liver tissues and serum samples were harvested 6 h after reperfusion. **(A)** Representative histological staining, Ly6G+ cells and CD11b+ cells infiltration and immunohistochemical staining of NLRP3 in ischemic livers. Scale bar: 30μm. **(B)** Suzuki’s histological score and sALT. **(C)** Quantitative analysis of infiltrated Ly6G+ cells and CD11b+ cells. **(D)** mRNA levels of IL-6, TNF-α and CXCL1, as well as mRNA levels of NLRP3 and IL-1β were determined using RT-qPCR. **(E)** Protein expressions of NLRP3, ASC, pro-caspase-1, cleaved caspase-1 p10, pro-IL-1β, IL-1β, PPARγ, Bcl-2, pro-caspase-3 and caspase-3 were detected using Western blot analysis. **(F)** Quantitative analysis of NLRP3 positively-stained cells. **(G)** ELISA analysis of IL-1β levels in animal serums. **(H)** Co-immunoprecipitation and Western blot for assessing the NLRP3 inflammasome assembly. The results are presented as the mean±SD of 4-6 animals per group. Blots shown are representative of 3 experiments with similar results. * *P* < 0.05 compared between the indicated groups. ** *P* < 0.01 compared between the indicated groups.

PPARγ has recently been regarded as an important regulator in inflammatory and immune responses besides its key role in energy metabolism [31, 32]. PPARγ can prevent monosodium urate (MSU)-induced NLRP3 inflammasome activation in HK-2 cells [33]. CGI-58-deficient macrophages exhibit mitochondrial dysfunction due to defective PPARγ signaling [34]. Consequently overproduced ROS potentiates secretion of IL-1β by activating NLRP3 inflammasome. Intriguingly, AA has recently been shown to enhance PPARγ expression in a dose-dependent manner in keloid fibroblasts [30]. Therefore, we assessed whether PPARγ involved in the protective effects of AA against I/R-induced inflammatory injury. As shown in Figure 2E, our results showed that AA led to a significant increase in PPARγ protein level compared to the controls, and this effect was even more significant in AA-treated I/R group. I/R alone also induced a moderate increase in PPARγ protein level compared to the controls, likely in a compensatory mechanism or a feedback loop. We then asked whether inhibition of PPARγ could offset the AA-mediated beneficial effects. To this end, GW9662, a PPARγ antagonist, was administered intraperitoneally 30 min prior to the treatment of AA. Although inhibition of PPARγ by GW9662 alone showed no significant impact on the parameters we measured, GW9662 abated the protective effects of AA on histopathological scorings and sALT levels (Figure 3A&3B). Consistently, GW9662 also attenuated the inhibitory effects of AA against infiltration of Ly6G+ neutrophils and CD11b+ monocytes/macrophages (Figure 3A&3C), as well as mRNA levels of IL-6, TNF-α and CXCL1 (Figure 3D), compared to the mice without GW9662 treatment. Furthermore, GW9662 reversed AA-mediated suppression of NLRP3 signaling, as evidenced by increase in mRNA levels of NLRP3 and IL-1β (Figure 3D), protein levels of NLRP3 and pro-IL-1β (Figure 3A, 3E&3F), formation of NLRP3-ASC-pro-caspase-1 complex (Figure 3H) as well as production of cleaved capase-1 p10 and mature IL-1β (Figure 3E). Due to an increased inflammatory levels associated with PPARγ inhibition, it was not surprising that GW9662 abolished the anti-apoptotic effects of AA (Figure 3E). Finally, I/R+GW9662 group was shown to be the worst in histological damage, inflammatory cell infiltration, as well as activation of NLRP3 inflammasome and apoptotic signaling pathway (Figure 3A-3G).

### Inactivation of KCs contributes to AA-induced protection against hepatic I/R injury

**Figure 4:**
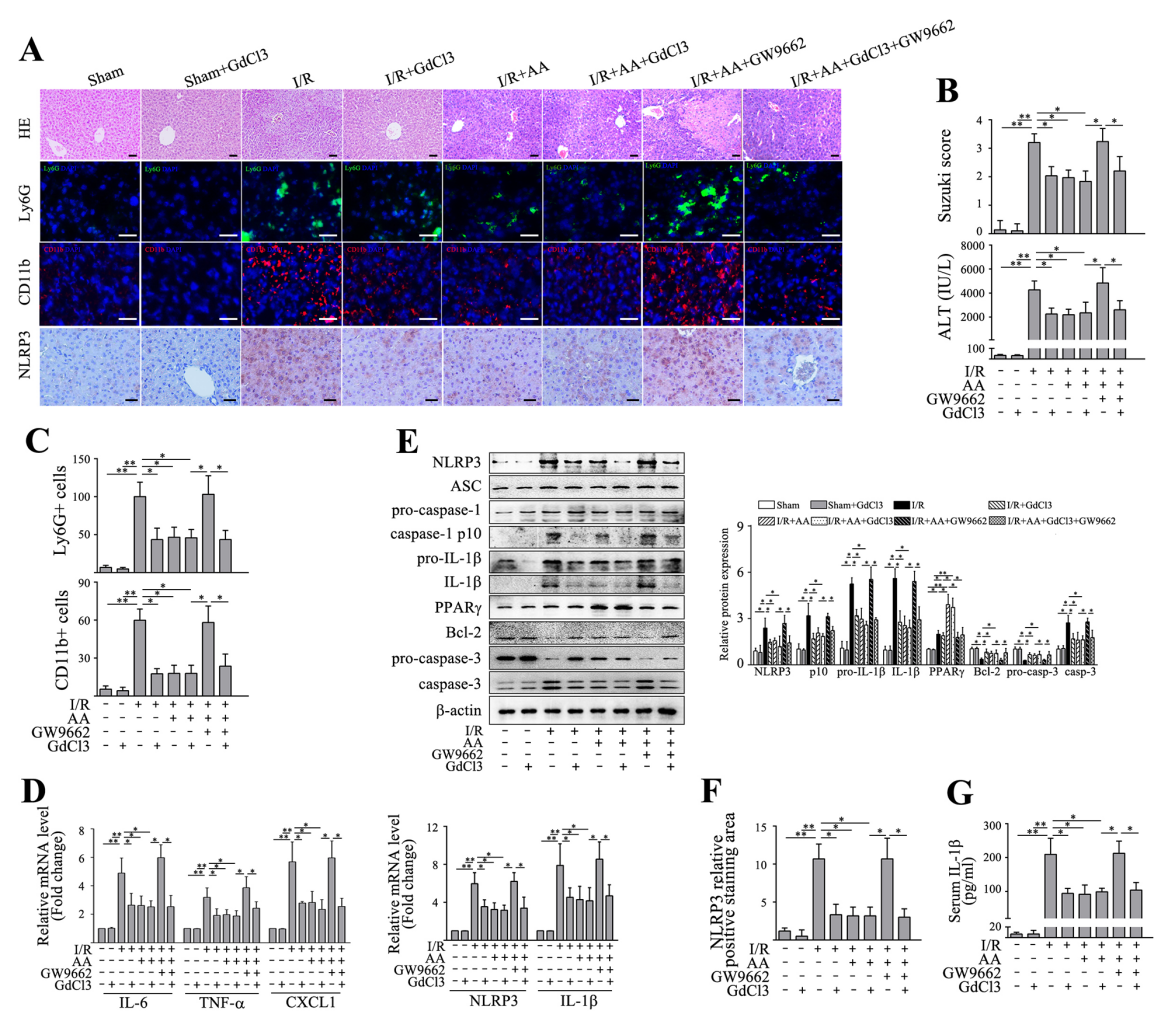
Inactivation of KCs contributes to AA-induced protection against liver I/R injury Mice were pretreated with GdCl3 24 h prior to AA treatment or AA+GW9662 treatment, followed by an I/R insult. Liver tissues and serum samples were harvested after 6 h reperfusion. **(A)** Representative histological staining, Ly6G+ cells and CD11b+ cells infiltration and immunohistochemical staining of NLRP3 in ischemic livers. Scale bar: 30μm. **(B)** Suzuki’s histological score and sALT. **(C)** Quantitative analysis of infiltrated Ly6G+ cells and CD11b+ cells. **(D)** mRNA levels of IL-6, TNF-α and CXCL1, as well as mRNA levels of NLRP3 and IL-1β were determined using RT-qPCR. **(E)** Protein expressions of NLRP3, ASC, pro-caspase-1, cleaved caspase-1 p10, pro-IL-1β, IL-1β, PPARγ, Bcl-2, pro-caspase-3 and caspase-3 were detected using Western blot analysis. **(F)** Quantitative analysis of NLRP3 positively-stained cells. **(G)** ELISA analysis of IL-1β levels in animal serums. The results are presented as the mean±SD of 4-6 animals per group. Blots shown are representative of 3 experiments with similar results. * *P* < 0.05 compared between the indicated groups. ** *P* < 0.01 compared between the indicated groups.

Recent findings have shown that KCs contribute to the major sources of NLRP3 inflammasome activation and IL-1β release in ischemic liver [3, 15, 35], despite the fact that all liver resident cells express certain levels of inflammasome molecules. We then asked which individual type of cells primarily contributed to AA-mediated inhibition of NLRP3. Hepatocytes and nonparenchymal cells (NPCs) fractions were firstly isolated from ischemic livers, and the mRNA levels of NLRP3 and IL-1β were then analyzed, respectively. As shown in Figure 2B, although I/R significantly increased mRNA expressions of NLRP3 and IL-1β compared to the controls in both hepatocytes and NPCs, the NPCs fraction had by far the highest NLRP3 and IL-1β expression. Treatment of AA further depressed this increase in NLRP3 and IL-1β mRNA level, a mechanism mainly operant in liver NPCs. Based on these findings and others, it is reasonable to assume that inactivation of KCs with an attenuated NLRP3 signaling accounted for the protective effects of AA against hepatic I/R injury. To confirm this, gadolinium chloride (GdCl3), a macrophage-suppressing agent widely used [3-5], was intravenously injected at 24 h prior to AA treatment or AA+GW9662 treatment, followed by an I/R insult. GdCl3 alone showed no significant impact on the measured parameters (Figure 4A-4G). Consistent with previous findings [3-5], we also found that GdCl3 acted to protect against hepatic I/R injury. However, no additional benefits of GdCl3 combined with AA treatment were observed when compared to AA-treated I/R group or GdCl3-treated I/R group, as indicated by similar histopathological scorings and sALT levels (Figure 4A&4B). GdCl3 is cytotoxic to macrophages, however, CD11b+ monocytes/macrophages has been shown to be spared from this effect [36]. Our data demonstrated that AA+GdCl3 did not further decrease infiltration of Ly6G+ neutrophils as well as CD11+ monocytes/macrophages compared to AA-treated I/R group (Figure 4A&4C). Consistently, AA+GdCl3 pretreatment showed no additional reduction in the mRNA levels of IL-6, TNF-α and CXCL1 compared to AA-treated I/R group (Figure 4D). Likewise, AA+GdCl3 did not decrease the mRNA levels and protein levels of NLRP3 and pro-IL-1β, as well as protein levels of cleaved caspase-1 p10 and mature IL-1β compared to AA-treated I/R group (Figure 4D-4G). Therefore, AA+GdCl3 had no additional benefits against apoptotic signaling compared to AA-treated I/R group (Figure 4E). In contrast, although GW9662 offset the protective effects of AA on hepatic I/R injury, these effects could be markedly reversed by GdCl3 pretreatment. As shown in Figure 4A-4E, I/R+AA+GdCl3+GW9662 group presented with an improvement in histopathological scoring and inflammatory injury compared to AA+GW9662-pretreated I/R group. Taken together, these findings suggested that inactivation of KCs by an attenuated NLRP3 signaling pathway contributed to the protective effects of AA against hepatic I/R injury.

### AA dampens NLRP3 inflammasome activation via PPARγ *in vitro*

**Figure 5:**
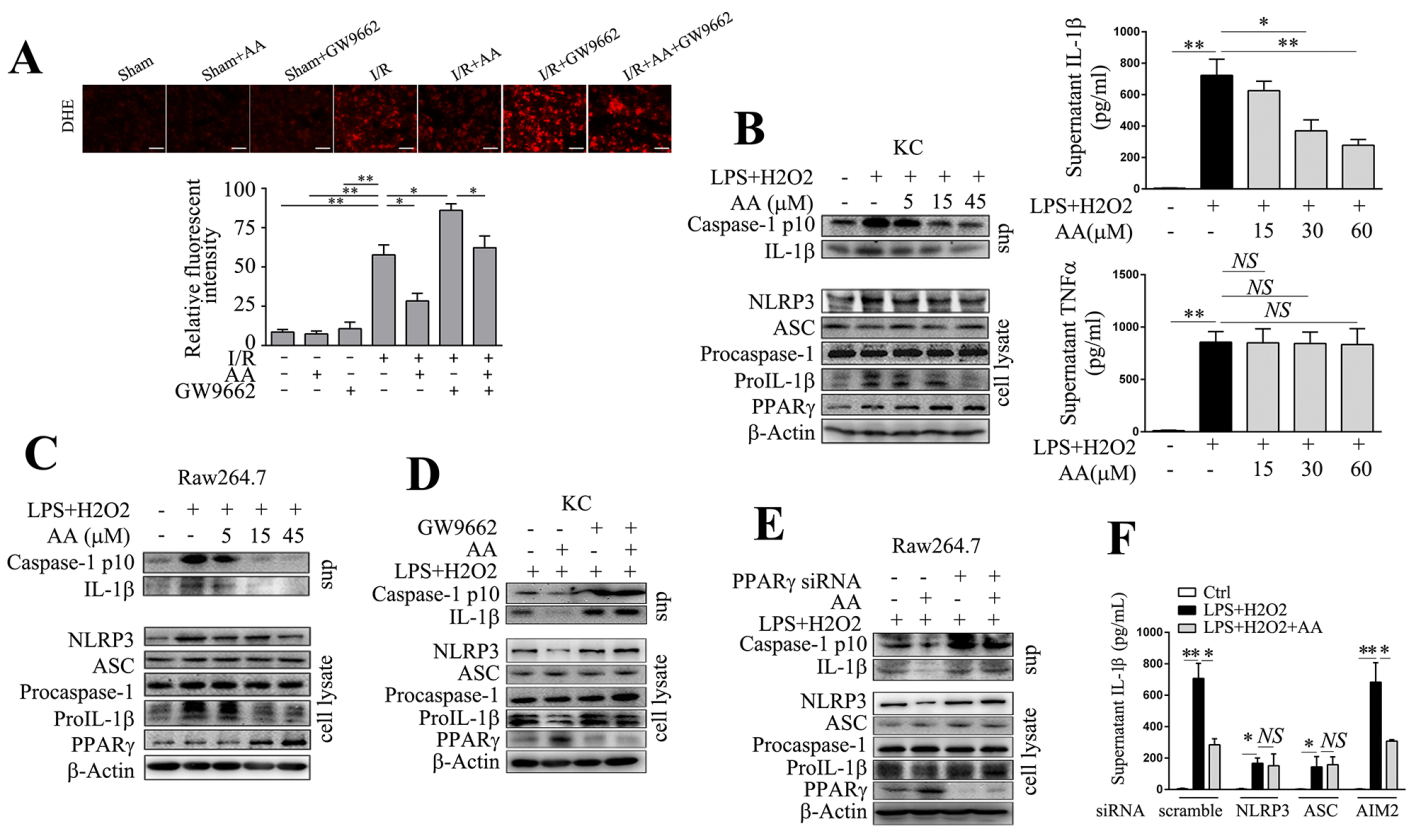
AA suppresses NLRP3 inflammasome activation via PPARγ *in vitro* **(A)** Representative sections of DHE-stained ischemic livers and quantitative analysis. Scale bar: 30μm. AA (5, 15, 45 μM, 1 h)-pretreated primary KCs **(B)** or RAW264.7 cells **(C)** were primed with LPS (100 ng/ml) for 4 h, followed by H_2_O_2_ (200 μM) for 2 h. Precipitated culture supernatants (sup) or cell lysates were used for detection of cleaved caspase-1 p10, IL-1β, NLRP3, ASC, pro-caspase-1, pro-IL-1β and PPARγ by Western blot analysis. IL-1β and TNF-α in culture supernatants were determined by ELISA. **(D)** KCs were pretreated with GW9662 (10 μM) for 30 min before AA (45 μM) or vehicle treatment, followed by LPS/H_2_O_2_ challenge. NLRP3 signaling molecules and PPARγ were assessed by Western blot analysis. **(E)** RAW264.7 cells were transfected with PPARγ siRNA for 48 h prior to AA or vehicle treatment, followed by LPS/H_2_O_2_ challenge. NLRP3 signaling molecules and PPARγ were assessed by Western blot analysis. **(F)** RAW264.7 cells were transfected with NLRP3 siRNA, ASC siRNA, AIM2 siRNA or scramble siRNA for 48 h prior to AA or vehicle treatment, followed by LPS/H_2_O_2_ challenge. IL-1β in culture supernatants were determined by ELISA. The results are representative of 3 experiments with similar results. *, ** Significant difference (*P* < 0.05, *P* < 0.01) compared between the indicated groups. *NS* No significant difference (*P* > 0.05) between the indicated groups.

Enhanced ROS generation represents one of the fundamental changes upon reintroduction of O2 to ischemia tissues. ROS production primarily from malfunctioning mitochondria may serve as “kindling” or triggering factor to activate NLRP3 inflammasome [37, 38]. To evaluate superoxide production, we analyzed dihydroethidium (DHE) staining of ischemic tissues under fluorescence microscopy. As shown in Figure 5A, I/R induced significant superoxide production, while AA counteracted this increase in a PPARγ-dependent manner. We further established an *in vitro* model by challenging primary KCs or RAW264.7 cells with LPS/H_2_O_2_. NLRP3 inflammasome activation requires two-step signaling, including priming (i.e. the activation of NF-κB pathway) and assembly [8]. Low dose of LPS priming could mimic the exposure to translocated pathogen-associated molecular patterns (PAMPs) that frequently derive from gut after hepatic I/R, and H_2_O_2_ challenge could further mimic the redox-induced injury. Both Western blot and ELISA analysis showed that AA treatment exhibited a concentration-dependent inhibition of IL-1β production in KCs induced by LPS/H_2_O_2_ (Figure 5B). Consistently, H_2_O_2_-induced activation of caspase-1 could be abolished by AA (Figure 5B). High concentration of AA also decreased protein levels of NLRP3 and pro-IL-1β. The downregulated NLRP3 signaling pathway induced by AA was correlated with increased expression of PPARγ. Secretion of TNF-α, which is independent of NLRP3 inflammasome, was not affected by AA treatment (Figure 5B). These effects observed in KCs could be basically reproduced in RAW264.7 cells (Figure 5C). In addition, as shown in Figure 5D&5E, either inhibition of PPARγ by GW9662 in KCs, or knockdown of PPARγ by a specific siRNA in RAW264.7 cells, abolished the inhibitory effect of AA on LPS/H_2_O_2_-induced NLRP3 activation. These findings suggested that AA depressed LPS/H_2_O_2_-induced NLRP3 inflammasome activation in a PPARγ-dependent manner. AIM2 inflammasome has also been reported to involve in hepatic I/R injury [3]. To rule out the possibility of its contribution to LPS/H_2_O_2_-induced IL-1β secretion, RAW264.7 cells were transfected with either scramble siRNA, or specific siRNA targeting NLRP3, ASC or AIM2, followed by LPS/H_2_O_2_ challenge. As shown in Figure 5F, knockdown of NLRP3 or ASC, but not AIM2, suppressed H_2_O_2_-induced secretion of IL-1β. In addition, AA pretreatment had no additional effect on IL-1β secretion after knockdown of NLRP3 or ASC, but not AIM2, further suggesting that AA inhibited LPS/H_2_O_2_-induced IL-1β secretion by targeting NLRP3 inflammasome.

### PPARγ/ROS/MAPK and PPARγ/ROS/NF-κB involve in AA-mediated suppression of NLRP3 inflammasome

**Figure 6:**
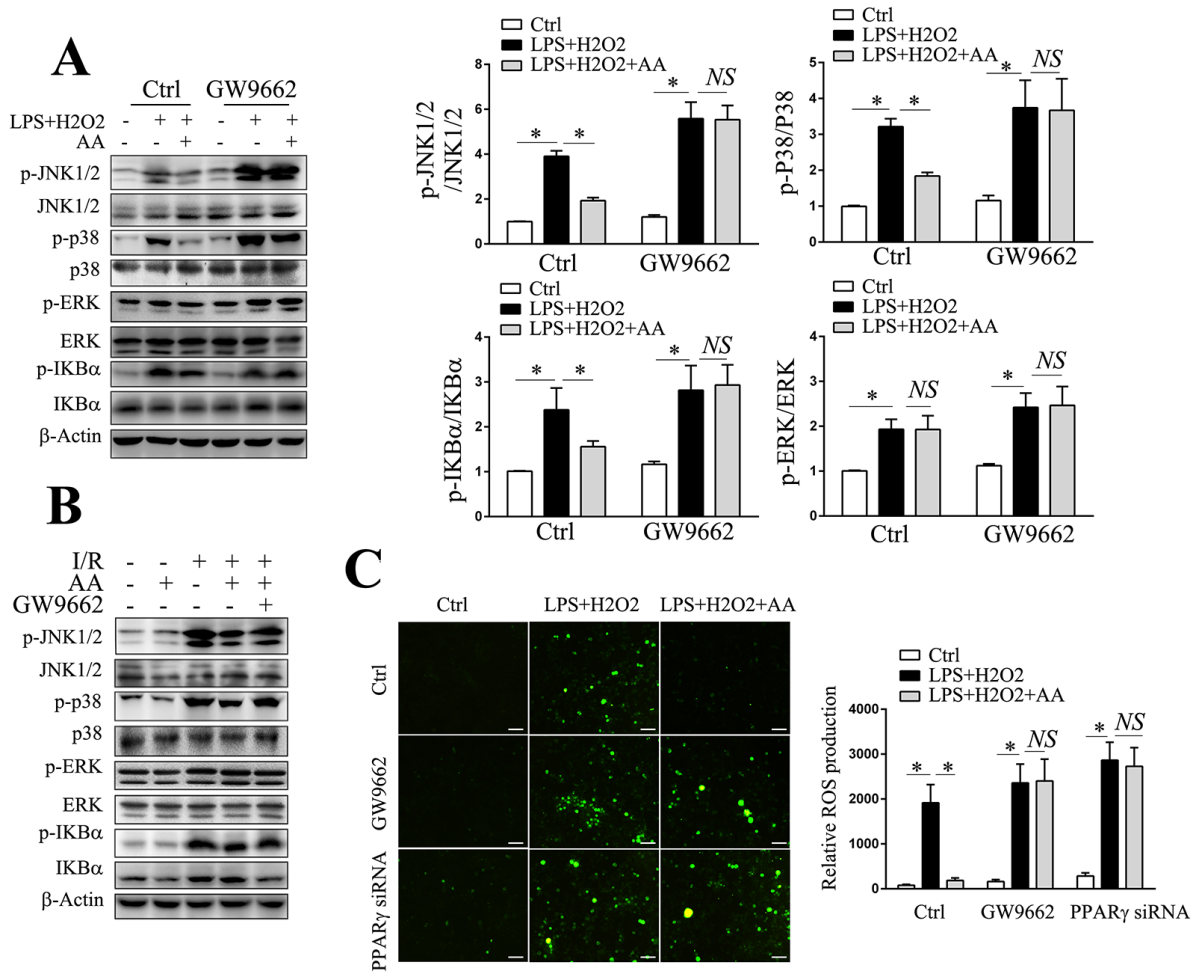
PPARγ/ROS/MAPK and PPARγ/ROS/NF-κB signaling pathways involve in AA-mediated suppression of NLRP3 inflammasome **(A)** KCs were pretreated with GW9662 for 30 min prior to AA or vehicle treatment, followed by LPS/H_2_O_2_ challenge. Cell lysates were used for detection of p-JNK, JNK, p-p38 MAPK, p38 MAPK, p-ERK, ERK, p-IκBα and IκBα by Western blot analysis. Quantities of phosphorylated forms are normalized to their total forms, respectively. **(B)** Liver lysates from the indicated groups were used for detection of p-JNK, JNK, p-p38 MAPK, p38 MAPK, p-ERK, ERK, p-IκBα and IκBα by Western blot analysis. **(C)** RAW264.7 cells transfected with PPARγ siRNA for 48 h prior to AA or vehicle treatment, or pretreated with GW9662 for 30 min before AA or vehicle treatment, were followed by LPS/H_2_O_2_ challenge. Intracellular ROS generation was measured using DCFH-DA. Scale bar: 30μm. The results are representative of 3 experiments with similar results. * Significant difference (*P* < 0.05) compared between the indicated groups. *NS* No significant difference (*P* > 0.05) between the indicated groups.

We also determined the effect of AA on ROS generation *in vitro* by using DCFH-DA. Consistent with the *in vivo* findings, AA was shown to inhibit the LPS/H_2_O_2_-induced production of ROS in RAW264.7 cells, an effect could either be abolished by GW9662 or transfection of PPARγ siRNA (Figure 6C). ROS are also proposed as central mediators in the processes of MAPKs and NF-κB activation, which can in turn regulate NLRP3 signaling pathway either at activation step or priming step [8, 39, 40]. Our results demonstrated that AA could reduce LPS/H_2_O_2_-elicited or I/R-elicited phosphorylation of JNK, p38 MAPK and IκBα, but not ERK, either *in vitro* or *in vivo*, respectively (Figure 6A&6B). The reductions in the phosphorylation levels of MAPKs and IκBα could further be reversed by GW9662. Therefore, these findings suggested that PPARγ/ROS/MAPK and PPARγ/ROS/NF-κB signaling pathway might involve in AA-mediated suppression of NLRP3 signaling pathway.

### AA blocks the detrimental effect of macrophage activation on hepatocyte viability and improves survival in a lethal hepatic I/R injury model

**Figure 7:**
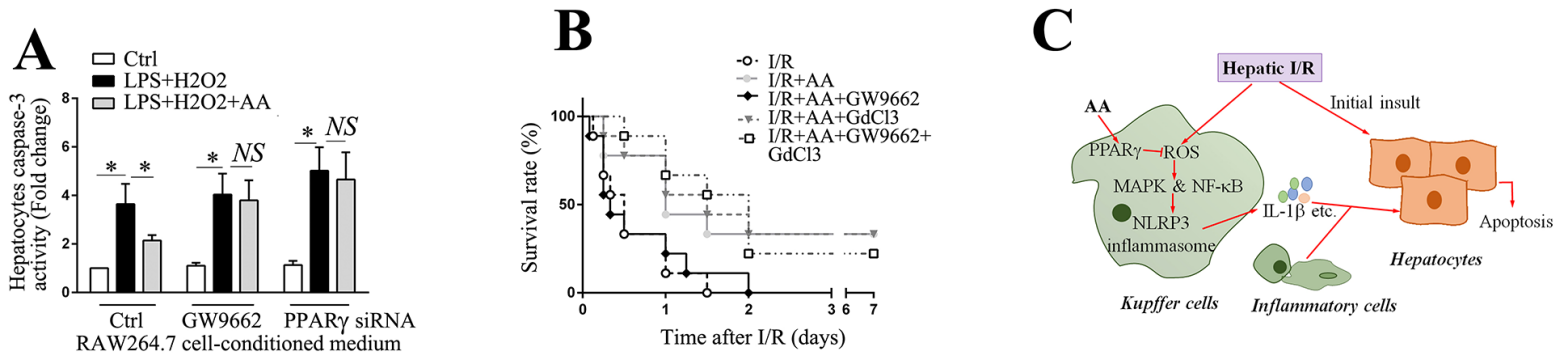
AA blocks the detrimental effect of macrophage activation on hepatocyte viability and improves survival in a lethal hepatic I/R injury model **(A)** RAW264.7 cells transfected with PPARγ siRNA for 48 h prior to AA, or pretreated with GW9662 for 30 min before AA, were followed by LPS/H_2_O_2_ challenge. Primary hepatocytes were then challenged with these various conditioned supernatants for 6 h prior to harvest for detection of casapse-3 activity. The results are representative of 3 experiments with similar results. * Significant difference (*P* < 0.05) compared between the indicated groups. *NS* No significant difference (*P* > 0.05) between the indicated groups. **(B)** A lethal hepatic I/R injury model was performed by surgical remove of non-ischemic shunt liver lobes at the end of 90 min ischemia, and the effect of AA on the survival benefit was followed for 7 days after surgery (n=9 of each group). **(C)** Schematic diagram for the protective effects of AA against hepatic I/R injury. AA leads to activation of PPARγ, which counteracts the KCs-mediated activation of NLRP3 inflammasome via down-regulation of ROS/MAPK and ROS/NF-κB signaling pathway during hepatic I/R, eventually reducing the levels of tissue injury.

Inflammatory mediators secreted from activated KCs can either directly or indirectly affect hepatocytes vitality [41]. To determine the effect of macrophage activation on the vitality of hepatocytes, primary cultured hepatocytes were challenged with the supernatants of RAW264.7 cells that were conditioned with different treatments, as indicated. The caspase-3 activity of hepatocytes was subsequently determined. As shown in Figure 7A, supernatants of RAW264.7 cells conditioned with LPS/H_2_O_2_ significantly increased caspase-3 activity of hepatocytes compared to those of controls, while supernatants from AA-treated RAW264.7 cells blunted the increase in caspase-3 activity of hepatocytes. This beneficial effect was markedly abolished either by the pretreatment of the LPS/H_2_O_2_-conditioned RAW264.7 cells with GW9662, or by the transfection with PPARγ siRNA. These data indicate that AA-induced suppression of NLRP3 inflammasome in RAW264.7 cells disrupts the detrimental cell-cell interaction and thereby protects hepatocytes against apoptosis. Finally, we investigated whether AA administration was accompanied by an improved survival in a lethal hepatic I/R model. As shown in Figure 7B, AA treatment significantly improved the survival from 0.0% to 33.3%. Treatment with GW9662 abolished the protective effects of AA. Pretreatment with GdCl3 further reversed the detrimental effect of GW9662 resulting from inhibition of PPARγ.

## DISCUSSION

Previous studies have demonstrated that AA attenuates focal cerebral I/R injury and myocardial I/R injury [21, 42]. AA has also been reported to be hepatoprotective in liver injury caused by CCl4, GalN/LPS, high-fat diet and Con A *in vivo* [23-26]. To our knowledge, however, there are no reports about its effects on hepatic I/R injury. Thus, the present study provided evidence that AA attenuated I/R-induced hepatic injury through attenuation of KCs activation via PPARγ/NLRP3 inflammasome signaling pathway.

Hepatic I/R injury contributes to significant morbidity and mortality in the clinical context, such as delayed liver graft function after a liver transplantation, liver failure in patients with shock and ischemic cholangiopathy as a long-term consequence [2]. NLRs are recently identified intracellular pattern recognition receptors (PRRs) that are essential to innate immune responses and tissue homeostasis. The importance of NLRP3 inflammasome in the pathogenesis of hepatic I/R has been supported by accumulating studies using various procedures, such as gene knockout, gene delivery, immunological neutralization and pharmacological inhibition procedures [6, 11-17]. Consistently, our data supported the key role of NLRP3 activation on the promotion of hepatic inflammatory injury post I/R. Administration of AA significantly suppressed NLRP3 activation and global inflammatory levels in the ischemic liver. The reductions of these inflammatory mediators appeared to facilitate the attenuation of the hepatic histopathological damage and apoptotic signaling. These findings were also confirmed by *in vitro* experiments in which AA pretreatment suppressed NLRP3 activation in LPS/H_2_O_2_-challenged KCs or RAW264.7 cells.

Activated KCs are the major sources of pro-inflammatory cytokines represented by TNF-α and IL-1β [1, 43]. The release of these initial cytokines further upregualtes their downstream targets including IL-6, IL-8, CD11/CD18, ICAM-1, MIP-2 and CXCL-1 [43]. These mediators in turn act to amplify Kupffer cell activation and promote neutrophil recruitment into the liver sinusoids. Although most cell types in the liver contribute to NLRP3 activation, KCs has shown by far the highest activity of NLRP3 inflammasome upon stimulation [3, 15, 35]. Consistent with this, our data showed that the NPCs fraction had a much higher expression of NLRP3 and IL-1β in the ischemic liver compared to hepatocytes fraction. Administration of AA further attenuated the increase in NLRP3 and IL-1β expression, a mechanism mainly operant in NPCs. Supporting this, KCs depletion by GdCl3 combined with AA had no additional benefits against hepatic I/R compared to AA-treated I/R group. These findings suggest that inactivation of KCs by an attenuated NLRP3 signaling pathway mainly contributes to the protective effects of AA against hepatic I/R injury. Interestingly, our data showed that AA only acted to reduce the production of TNF-α *in vivo* but not *in vitro*. There are several possibilities that may account for these seemly contradictory findings. Firstly, compared to the *in vitro* models, it is more difficult to exclude the indirect influences from the complex network of cytokines and cell-cell interactions *in vivo*, and focus on the definitive effect of AA on TNF-α production. Secondly, the *in vitro* model we used currently was different from any previous studies related to AA. We employed a two-step activation model, with the mediums being changed before H_2_O_2_ challenge. In addition, previous study has shown that AA only inhibited TNF-α production in a certain concentration range (60-120μM) in response to LPS [19]. It is possible that AA may show more specificity on the inhibition of NLRP3 inflammasome rather than on that of TNF-α, when a lower concentration range of AA combined with an inflammasome activation model was set. Similarly to us, previous studies have demonstrated that another plant extract resveratrol presented with more specificity on NLRP3 over TNF-α in a certain concentration range despite an attenuated NF-κB signaling [44, 45].

PPARγ belongs to PPARs family and is ubiquitously expressed in a wide ranges of tissues. Previous studies have shown a key role of PPARγ in energy metabolisms. Besides this, emerging effects of PPARγ have also been reported including anti-inflammatory potentials especially [31, 32]. Activation of PPARγ has been shown to inhibit the release of various cytokines including IL-1β [46]. In hepatic I/R injury, PPARγ upregulation is proposed to be a key mechanism of the benefits of different pharmacological or surgical strategies. Activation of PPARγ by pioglitazone inhibits hepatocytes apoptosis and significantly improved the survival of mice in a lethal model of hepatic I/R injury [46]. PPARγ deficient mice displays more severe inflammatory injuries than wild-type mice under warm ischemia conditions [47]. Consistently, our data showed that the protective effect of AA against hepatic I/R injury was correlated with increased expression of PPARγ. This protective effect appears dependent on PPARγ, since pharmacological inhibition of PPARγ by GW9662 abolished the beneficial effects of AA against NLRP3 inflammasome activation and hepatic I/R injury. Consistently, either GW9662 or PPARγ knockdown abolished the inhibitory effect of AA on LPS/H_2_O_2_-induced NLRP3 inflammasome activation *in vitro*. In agreement with our findings, previous study has also shown an inhibitory effect of PPARγ against NLRP3 activation by using a model of MSU-challenged HK-2 cells [33]. Moreover, CGI-58-deficient macrophages have shown to exhibit robust activation of NLRP3 inflammasome due to defective PPARγ signaling [34].

NLRP3 inflammasome activation requires two-step signaling, including priming and assembly [8]. Mitochondrial dysfunction acts upstream of NLRP3 activation by providing ROS and oxidized mitochondrial DNA to trigger NLRP3 oligommerization [37, 38]. Almost all known NLRP3 activators induce the production of short-lived ROS, and ROS scavengers in turn ameliorate the activation of NLRP3 in response to different agonists [38]. Consistently, we showed that overproduction of ROS either by I/R or LPS/H_2_O_2_ challenge was correlated with the extent of NLRP3 activation. This effect could be greatly abolished by treatment of AA via a PPARγ-dependent manner. Supporting this, PPARγ agonist pioglitzone has been previously shown to increase the oxygen consumption and mitochondrial DNA contents, and to induce the expression of various factors involved in mitochondrial biogenesis such as mitochondrial transcription factors A (TFAM) [48, 49]. PPARγ also acts to increase mitochondrial membrane potential, induce cytochrome c oxidase (CytOx)-I and CytOxIV expression levels, upregulate mitochondrial uncoupling protein 2, restore mitochondrial complex I activity and prevent cell death [48, 50].

Activation of MAPKs and NF-κB signaling further act to upregulate NLRP3 signaling either at priming step or activation step [8, 39, 40]. Among mammalian MAPKs, ERKs are activated by mitogenic and proliferative stimuli, whereas p38 and JNK are activated by a variety of cellular stresses. p38 and JNK are phosphorylated and activated several minutes after reperfusion [51]. Treatment with p38 or JNK activators shows increased transaminase levels and necrosis in hepatic I/R model, while MAPK inhibition reduced I/R injury [52, 53]. NF-κB is a transcription factor that regulates expression of inflammatory cytokines and cellular stress. Phosphorylation of IκBα dissociates from NF-κB and predispose NF-κB to undergo nuclear translocation. Because lower generation of ROS resulted from a higher PPARγ expression by AA, it is not surprising that JNK, p38 MAPK and NF-κB signaling pathway were also suppressed, either in response to I/R or to LPS/H_2_O_2_ challenge. Therefore, AA attenuated NLRP3 signaling both at expression level and assembly level.

Although our data described a model whereby AA-mediated PPARγ upregulation repressed NLRP3 signaling and protected against hepatic I/R injury, several limitations should be taken into account on the data interpretation. Firstly, based on the versatile activities of AA, our model may only partially explain its protective effect against hepatic I/R. Previous studies have shown that AA acted to regulate various downstream pathways such as Nrf2, AMPK, mTOR, Akt/GSK-3β/HIF-1α and miR-1290/HIF3A/HIF-1α, among many others [42, 54-56]. Given that most of these pathways are critically involved in I/R, it is probable that AA may mediate hepatoprotection through these pathways besides PPARγ/NLRP3 axis. Secondly, the regulation mechanisms between PPARγ, ROS, MAPK/NF-κB, and NLRP3 haven’t been fully elucidated. As a well-known transcription factor, PPARγ can modulate gene expression by binding directly to specific PPAR-response elements (PPRE) in target genes as heterodimers with the retinoid X receptors (RXRs) [57]. Our sequence analysis also demonstrates that the promoter region of NLRP3 contains several potential PPRE motifs either in DR1 (AGGTCA N AGGTCA) or DR2 (AGGTCA NN AGGTCA) format, with mismatched nucleotides being less than 3. PPARγ also acts to repress proinflammatory gene expression in a ligand-dependent manner by antagonizing the activities of other transcription factors such as members of NF-κB and activator protein-1 (AP-1) families [58, 59]. In addition, PPARγ has been shown to have E3 ubiquitin ligase activity and induce degradation of p65 [60]. Thus, further experiments are still required to elucidate that whether these mechanisms are involved in AA-mediated inhibition of NLRP3 signaling in hepatic I/R.

In conclusion, we identified a key mechanism underlying the hepatoprotective properties of AA (Figure 7C). This mechanism involves the activation of PPARγ, which counteracts the KCs-mediated activation of NLRP3 inflammasome via down-regulation of ROS/MAPK and ROS/NF-κB signaling pathway during hepatic I/R, eventually reducing the levels of tissue injury. Our data provide important experimental data for further pharmacological research, and a rationale for future clinical trials and applications.

## MATERIALS AND METHODS

### Animals

Male C56BL/6 (8-10 weeks, 20-25 g weight) were purchased from Shanghai Laboratory Animal Center (SLAC, Shanghai, China). All animals were maintained under specific pathogen free condition at constant room temperature (21 ± 2°C) and allowed to water and food *ad libitum* in 12 h dark/light cycle. The mice were kept at least 1 week in animal house before performing any experiment. The experimental protocol was approved by the Institutional Animal Care and Use Committee of Jiangsu University in China. All animal experiments were conducted in conformance with the Guide for the Care and Use of Laboratory Animals published by the US National Institutes of Health (NIH Publication, 8^th^ edition, 2011).

### Chemicals and reagents

Culture medium DMEM, penicillin, streptomycin, fetal bovine serum (FBS) and Trypsin-EDTA were purchased from Gibico, USA. Percoll, LPS, 30% (w/w) H_2_O_2_, GW9662 and AA were purchased from Sigma-Aldrich, USA. Lipofectamine 2000 and TRIzol Reagent were obtained from Life Technologies, USA. Alexa Fluor 488-conjugated anti-Ly6G, Alexa Fluor 488-conjugated anti-F4/80 and Alexa Fluor 594-conjugated anti-CD11b were purchased from Bioss, China. Monoclonal anti-mouse NLRP3, caspase-3 and β-actin were from Cell Signaling Technology, USA. Polyclonal anti-mouse ASC, caspase-1, IL-1β and PPARγ were from Santa Cruz Biotechnology. Polyclonal anti-mouse c-Jun N-terminal kinase (JNK), p-JNK, extracellular signal-regulated kinase (ERK), p-ERK, p38 mitogen-activated protein kinase (MAPK), p-p38 MAPK, nuclear factor kappa B (NF-κB) inhibitor alpha (IκBα) and p-IκBα were purchased from Sangon Biotech, China. Polyclonal anti-mouse B-cell lymphoma 2 (Bcl-2) was from Proteintech, China. 4′,6-diamidino-2-phenylindole (DAPI) was obtained from Life Technologies. Enzyme-linked immunosorbent assay (ELISA) kits for IL-1β and tumor necrosis factor (TNF)-α were purchased from BD Biosciences, USA. Protein A/G-agarose beads were purchased from Santa Cruz Biotechnology. 2’-7’-Dichlorodihydrofluorescein diacetate (DCFH-DA) and Caspase-3 Colorimetric Assay Kit were purchased from Beyotime Institute of Biotechnology, China. Bicinchoninic acid (BCA) Protein Assay kit was obtained from Pierce Biotechnology, USA. ECL system was purchased from Vazyme Biotech, China. Clean-Blot™ IP Detection Reagent (HRP) kit was purchased from Thermo Scientific, USA. 3-amino-9-ethylcarbazole (AEC) kit was purchased from BOSTER, China. Alanine Aminotransferase (ALT) Assay Kit and Aspartate Aminotransferase (AST) Assay Kit were purchased from Jiancheng Bioengineering Institute, China. One Step PrimeScript TM RT-PCR kit and SYBRR Premix-Ex TaqTM Kit were purchased from Takara, Japan.

### Mouse liver I/R injury model

The mice underwent hepatic I/R surgery or sham operations. Partial hepatic ischemia was induced as previously described [61]. In brief, mice were anesthetized by injection intraperitoneally (i.p.) with sodium pentobarbital (60mg/kg), and injected with heparin (100 U/kg). A midline laparotomy was performed and an atraumatic clip was placed across the portal vein, hepatic artery, and bile duct to interrupt blood supply to the left lateral and median lobes (∼70%) of the liver. After 60 min of partial hepatic ischemia, the clip was removed to initiate reperfusion. Sham mice underwent the same protocol without vascular occlusion. Mice were maintained on a heating pad (37°C) to avoid temperature fall during the whole procedure. At the 6 h after reperfusion, mice were sacrificed, and samples of blood and ischemic lobes were collected for analyses. In some mice, to assess animal survival, the non-ischemic shunt liver lobes were surgically removed at the end of 90 min ischemia, so that survival was dependent on the function of the liver tissue subjected to ischemic liver, as described previously [46]. Mice were then followed for 7 days after surgery.

### Drug treatment

AA (30 mg/kg body weight, dissolved in 0.1% DMSO saline solution) was given by oral gavage 1 h before hepatic I/R surgery. The dosage chosen was based on previous studies as well as our preliminary experiments to ensure both the efficacy and safety. Equivalent volume of 0.1% DMSO saline solution served as a vehicle. To assess the possible involvement of PPARγ in the protective effect of AA, GW9662 (2 mg/kg body weight, dissolved in 0.1% DMSO saline solution), a specific PPARγ antagonist, was pre-administered via i.p. 0.5 h before AA treatment and then hepatic I/R was performed. For depletion of KCs, gadolinium chloride (GdCl3) (20 mg/kg body weight, dissolved in phosphate buffered saline (PBS) was administered to mice via the tail vein 24 h prior to surgery. Equivalent volume of PBS served as a vehicle. As confirmed by immunofluorescent staining with Alexa Fluor 488-conjugated anti-F4/80 antibody, depletion of KCs by GdCl3 was greater than 75%.

### Isolation of primary hepatocytes, NPCs and KCs

For quantification of NLRP3 and IL-1β expression in different cell types after I/R, primary hepatocytes and NPCs were isolated from ischemic livers of C57BL/6 mice, as indicated. Briefly, the mice livers were perfused with Ca2+ and Mg2+-free Hank’s buffered salt solution (HBSS) containing EGTA (2.5 mM) via portal vein and then they were perfused again with 0.05% collagenase IV HBSS solution. Digested livers were dissected and then gently teased with forceps until they were in solution. The cell suspensions were filtered through a 100-um nylon cell strainer. NPCs were separated from the hepatocytes by one cycle of differential centrifugation (400 rpm for 5 min). The supernatant was centrifuged further (400 rpm for 5 min and two cycles of 1500 rpm for 5 min) to obtain NPCs. The NPCs did not contain hepatocytes, as assessed by light microscopy. Total RNA was extracted from the hepatocytes fraction and the NPCs fraction, respectively.

Alternatively, for primary cell culture, the hepatocytes and NPCs suspensions were obtained as described above except that livers from normal C57BL/6 mice were used. The hepatocytes suspensions were then centrifuged using 25% Percoll for 5 min at 800 rpm with the brake option off. The pellets were washed with DMEM supplemented with 10% FBS, and then cells were seeded into a collagen pre-coated 100 mm tissue culture plates. After 24 h, non-adherent cells were removed by aspiration, and fresh media were added. Hepatocytes purity exceeded 98%, as assessed by light microscopy, and viability typically was 95%, as determined by trypan blue exclusion assay. For KCs culture, the NPCs supernatants were layered onto a 50/25% two-step Percoll gradient for 15 min at 3200 rpm with brake option off. KCs in the middle layer were collected and washed with DMEM supplemented with 10% FBS. Three hours following seeding, non-adherent cells were removed by replacing the culture medium. The purity of KCs was greater than 90%, as quantified with Alexa Fluor 488-conjugated anti-F4/80 immunostaining.

### Cell culture

The murine macrophage cell line, RAW264.7 cell was obtained from Shanghai Institute of Cell Biology (Shanghai, China) and cultured in DMEM containing 10% FBS and a 100 U/ml penicillin/streptomycin mixture. Cells with passage numbers less than 20 were used. RAW264.7 cells, as well as primary hepatocytes and KCs were cultured at 37 °C with 100% humidity in 5% CO2 using standard cell culture techniques.

### Inflammasome activation

RAW264.7 cells or primary KCs were incubated with AA (5, 15, 45 μM) or vehicle for 1 h before being primed with LPS (100 ng/ml) for 4 h in serum-free DMEM medium. The cells were washed twice with medium and were then challenged with H_2_O_2_ (200 μM) for 2 h. To assess the possible involvement of PPARγ in AA-mediated inhibition of NLRP3 inflammasome, cells were either pretreated with GW9662 (10 μM) 1h before LPS priming, or transfected with PPARγ siRNA 48 h before LPS priming, followed by H_2_O_2_ challenge, as indicated. To determine the role of NLRP3 inflammasome on LPS/H_2_O_2_-induced IL-1β release, cells were transfected with NLRP3 siRNA, ASC siRNA, AIM2 siRNA or scramble siRNA 48 h before LPS priming, followed by H_2_O_2_ challenge, as indicated. After the treatments, cell pellets and cell supernatants were collected for Western blotting or ELISA.

### RNA isolation and reverse transcription quantitative polymerase chain reaction (RT-qPCR)

Expression of NLRP3, IL-1β, TNF-α, IL-6 and chemokine (C-X-C motif) ligand (CXCL)-1 in ischemic livers and/or isolated cells was analyzed by RT-qPCR. Briefly, total RNA was extracted with TRIzol Reagent and cDNA synthesis was performed with a One Step PrimeScript TM RT-PCR kit using 2.5 μg total RNA. Quantitative PCR analysis was carried out using the SYBR Premix-Ex Tag Kit on an ABI Prism 7300 Sequence Detection System (Applied Biosystems, CA). The primer sequences are listed as follows: TNF-α forward: 5’-TGGAACTGGCAGAAGAGGCACT-3’, reverse: 5’-GTTCAGTAGACAGAAGAGCGTGGTG-3’, IL-1β forward: 5’-CACTACAGGCTCCGAGATGAACAAC-3’, reverse: 5’-TTGTCGTTGCTTGGTTCTCCTTGT-3’, NLRP3 forward: 5’-TGCGATCAACAGGCGAGACCT-3’, reverse: 5’-CCATCCACTCTTCTTCAAGGCTGTC-3’, CXCL1 forward: 5’-CTGCACCCAAACCGAAGTC-3’, reverse: 5’- AGCTTCAGGGTCAAGGCAAG-3’, IL-6 forward: 5’-TACCACTTCACAAGTCGGAGGCTTA-3’, reverse: 5’-CTGCAAGTGCATCATCGTTGTTCAT-3’, β-actin forward: 5’-GTGATGGTGGGAATGGGTCAGAAG -3’, reverse: 5’-CATTGTAGAAGGTGTGGTGCCAGAT-3’. Each gene expression was normalized to β-actin before the fold change was calculated.

### RNA-mediated interference

For RNA-mediated interference, cells were transfected with 40 nM validated mouse PPARγ siRNA, NLRP3 siRNA, ASC siRNA, AIM2 siRNA or scramble siRNA (GenePharma, Shanghai) using Lipofectamine 2000 transfection reagent. The sequences of siRNA were listed as follows: 5’-GACAUGAAUUCCUUAAUGAUU-3’ for PPARγ, 5’-CAUCAAUGCUGCUUCGACAUU-3’ for NLRP3, 5’-GAGCAGCUGCAAACGACUAUU-3’ for ASC, 5’-ACAUAGACACUGAGGGUAU-3’ for AIM2, and 5’-AAUUCUCCGAACGUGUCACGU-3’ as scramble siRNA. Effective knockdown by each targeted siRNA was confirmed by Western blot, with an efficiency approximately 70%. Forty-eight hours after transfection, the cells were ready for the following experiments.

### Measurement of cytokine levels

The levels of IL-1β and TNF-α in the serums and in the culture mediums were measured using commercially available ELISA kits according to the manufacturer’s instructions.

### Measurement of serum parameters

Serum ALT (sALT) and serum AST (sAST) were measured using corresponding diagnostic kits according to the manufacturers’ instructions.

### Western blotting

Protein samples from liver homogenates or cell lysates were quantified using the BCA Protein Assay kit. Equal amounts of protein samples were loaded on 8-15% polyacrylamide gels, separated by SDS-PAGE, and then transferred to nitrocellulose membranes. Bands were probed immunologically using anti-mouse NLRP3 (1:1000), ASC (1:500), caspase-1 (1:500), IL-1β (1:500), PPARγ (1:1000), Bcl-2 (1:1000), caspase-3 (1:1000), p-JNK (1:1000), JNK (1:1000), p-p38 (1:1000), p38 (1:1000), p-EKR (1:1000), ERK (1:1000), p-IκBα (1:1000), IκBα (1:1000) or β-actin (1:5000). Signals were detected using an ECL system according to the manufacturer’s instructions. Intensities of the immunoreactive bands were determined using Image LabTM software (version 4.1, Bio-Rad, CA).

### Immunoprecipitation

To assess the association of proteins in the inflammasome, 600 μg of liver lysates were pre-cleared with protein A/G-agarose beads and then incubated overnight with anti-ASC antibody (1:500) or normal IgG (1:500) at 4°C overnight with rotary agitation. The immune complexes were then precipitated via incubation with protein A/G-agarose beads for 6 h followed by extensive washing with cold PBS. Immunoprecipitated proteins were eluted with 2 × SDS loading buffer and separated using SDS gels. Bands were probed immunologically using anti-mouse NLRP3 and caspase-1, and were detected using Clean-Blot™ IP Detection Reagent (HRP) kit according to the manufacturer’s instructions.

### Histology, immunohistochemistry and immunofluorescence

For histology, the ischemic livers were perfused with saline and then were fixed with 10% (w/v) formalin in PBS and embedded in paraffin. The paraffin-embedded samples were cut into 4-mm-thick sections and stained with H&E. The severity of I/R injury was graded using Suzuki’s criteria on a scale from 0-4 [62]. For immunohistochemistry, tissue sections were deparaffinised with xylene and stepwise rehydrated with serial dilutions of ethanol. Antigen retrieval was performed by incubating the sections in antigen retrieval buffer for 15 minutes at 97 °C. After antigen retrieval, the sections were incubated with 3% H_2_O_2_ for 15 min at room temperature, then with 3% normal goat serum at 37 °C for 20 min and finally with anti-NLRP3 antibody (1:200) overnight at 4 °C. The sections were subsequently washed twice in PBS and incubated with a biotinylated secondary antibody for 30 min at 37 °C. The sections were then washed again, incubated with streptavin-peroxidase complex for 30 min at 37 °C, and washed once more in PBS. Colorization was monitored using an AEC kit. Finally, the sections were counterstained with hematoxylin, dehydrated and mounted with resinene and examined microscopically (×200). For each animal, more than five tissue sections, including representative sections, were analyzed. For immunofluorescence, frozen 4 um-thick sections of ischemic livers were incubated with 0.3% Triton X-100 and 3% normal goat serum for 30 min, the sections were incubated with Alexa Fluor 488-conjugated anti-mouse Ly6G, or with Alexa Fluor 594-conjugated anti-mouse CD11b overnight at 4 °C. DAPI was used for nuclear counterstaining. The samples were observed under a fluorescence microscope (Leica, Mannheim, Germany). Positive cells were counted blindly in 10 high power field (HPF)/section (×200).

### Detection of ROS *in situ*

DHE was used to measure the superoxide production in ischemic tissues as described earlier [63, 64]. Briefly, transverse sections of frozen tissues were incubated with DHE (10μM) in a dark, humidified container at 37°C for 30min. The dye was excited at 480 nm, and the emission was detected at 580 nm by fluorescence microscope. Images were quantified by fluorescence intensity using ImageJ (National Institutes of Health, MD, USA).

### Detection of ROS *in vitro*

Intracellular ROS generation was measured by using DCFH-DA according to the manufacturer’s instructions. Briefly, at the end of treatments, RAW264.7 cells were incubated with DCFH-DA (10μM) for 30 min at 37 °C in the dark. The dye was excited at 488 nm, and the emission was detected at 525 nm by fluorescence microscope.

### Determination of caspase-3 activity

As described in *inflammasome activation* section, RAW264.7 cells were pre-transfected with PPARγ siRNA or pretreated with GW9662 prior to AA or vehicle treatment, followed by LPS/H_2_O_2_ challenge. After that, the conditioned mediums were collected for challenging primary hepatocytes, respectively. After incubation for 6 h, the hepatocytes were harvested for detection of caspase-3 activity according to the manufacturer’s protocol.

### Statistical analysis

All statistical analysis were conducted with SPSS^®^ version 17.0 (SPSS, Chicago, USA). Differences between two dependent groups were evaluated with the paired Student’s t-test. Comparisons among multiple groups were performed with one-way ANOVA followed by Bonferroni post-hoc tests. Animal survival analysis was performed with the Kaplan-Meier analysis and the log-rank test. All *P*-values were two-tailed, and *P* < 0.05 was accepted as being statistically significant. GraphPad Prism 6.0 (La Jolla, CA, USA) was used to generate the graphs.

## SUPPLEMENTARY MATERIALS FIGURE



## References

[1] Klune JR, Tsung A (2010). Molecular biology of liver ischemia/reperfusion injury: established mechanisms and recent advancements. Surg Clin North Am.

[2] Zhai Y, Petrowsky H, Hong JC, Busuttil RW, Kupiec-Weglinski JW (2013). Ischaemia-reperfusion injury in liver transplantation--from bench to bedside. Nat Rev Gastroenterol Hepatol.

[3] Kim HY, Kim SJ, Lee SM (2015). Activation of NLRP3 and AIM2 inflammasomes in Kupffer cells in hepatic ischemia/reperfusion. FEBS J.

[4] Liu P, McGuire GM, Fisher MA, Farhood A, Smith CW, Jaeschke H (1995). Activation of Kupffer cells and neutrophils for reactive oxygen formation is responsible for endotoxin-enhanced liver injury after hepatic ischemia. Shock.

[5] Hisama N, Yamaguchi Y, Ishiko T, Miyanari N, Ichiguchi O, Goto M, Mori K, Watanabe K, Kawamura K, Tsurufuji S, Ogawa M (1996). Kupffer cell production of cytokine-induced neutrophil chemoattractant following ischemia/reperfusion injury in rats. Hepatology.

[6] Kamo N, Ke B, Ghaffari AA, Shen XD, Busuttil RW, Cheng G, Kupiec-Weglinski JW (2013). ASC/caspase-1/IL-1beta signaling triggers inflammatory responses by promoting HMGB1 induction in liver ischemia/reperfusion injury. Hepatology.

[7] Szabo G, Petrasek J (2015). Inflammasome activation and function in liver disease. Nat Rev Gastroenterol Hepatol.

[8] Gross O, Thomas CJ, Guarda G, Tschopp J (2011). The inflammasome: an integrated view. Immunol Rev.

[9] Takeuchi D, Yoshidome H, Kato A, Ito H, Kimura F, Shimizu H, Ohtsuka M, Morita Y, Miyazaki M (2004). Interleukin 18 causes hepatic ischemia/reperfusion injury by suppressing anti-inflammatory cytokine expression in mice. Hepatology.

[10] Suzuki S, Toledo-Pereyra LH (1994). Interleukin 1 and tumor necrosis factor production as the initial stimulants of liver ischemia and reperfusion injury. J Surg Res.

[11] Ouzounidis N, Giakoustidis A, Poutahidis T, Angelopoulou K, Iliadis S, Chatzigiagkos A, Zacharioudaki A, Angelopoulos S, Papalois A, Papanikolaou V, Giakoustidis D (2016). Interleukin 18 binding protein ameliorates ischemia/reperfusion-induced hepatic injury in mice. Liv Transpl.

[12] Shito M, Wakabayashi G, Ueda M, Shimazu M, Shirasugi N, Endo M, Mukai M, Kitajima M (1997). Interleukin 1 receptor blockade reduces tumor necrosis factor production, tissue injury, and mortality after hepatic ischemia-reperfusion in the rat. Transplantation.

[13] Furuichi K, Wada T, Iwata Y, Kokubo S, Hara A, Yamahana J, Sugaya T, Iwakura Y, Matsushima K, Asano M, Yokoyama H, Kaneko S (2006). Interleukin-1-dependent sequential chemokine expression and inflammatory cell infiltration in ischemia-reperfusion injury. Crit Care Med.

[14] Harada H, Wakabayashi G, Takayanagi A, Shimazu M, Matsumoto K, Obara H, Shimizu N, Kitajima M (2002). Transfer of the interleukin-1 receptor antagonist gene into rat liver abrogates hepatic ischemia-reperfusion injury. Transplantation.

[15] Huang H, Chen HW, Evankovich J, Yan W, Rosborough BR, Nace GW, Ding Q, Loughran P, Beer-Stolz D, Billiar TR, Esmon CT, Tsung A (2013). Histones activate the NLRP3 inflammasome in Kupffer cells during sterile inflammatory liver injury. J Immunol.

[16] Zhu P, Duan L, Chen J, Xiong A, Xu Q, Zhang H, Zheng F, Tan Z, Gong F, Fang M (2011). Gene silencing of NALP3 protects against liver ischemia-reperfusion injury in mice. Hum Gene Ther.

[17] Shimizu S, Eguchi Y, Kamiike W, Akao Y, Kosaka H, Hasegawa J, Matsuda H, Tsujimoto Y (1996). Involvement of ICE family proteases in apoptosis induced by reoxygenation of hypoxic hepatocytes. Am J Physiol.

[18] Pittella F, Dutra RC, Junior DD, Lopes MT, Barbosa NR (2009). Antioxidant and cytotoxic activities of Centella asiatica (L) Urb. Int J Mol Sci.

[19] Yun KJ, Kim JY, Kim JB, Lee KW, Jeong SY, Park HJ, Jung HJ, Cho YW, Yun K, Lee KT (2008). Inhibition of LPS-induced NO and PGE2 production by asiatic acid via NF-kappa B inactivation in RAW 264.7 macrophages: possible involvement of the IKK and MAPK pathways. Int Immunopharmacol.

[20] Won JH, Shin JS, Park HJ, Jung HJ, Koh DJ, Jo BG, Lee JY, Yun K, Lee KT (2010). Anti-inflammatory effects of madecassic acid via the suppression of NF-kappaB pathway in LPS-induced RAW 264.7 macrophage cells. Planta Med.

[21] Lee KY, Bae ON, Serfozo K, Hejabian S, Moussa A, Reeves M, Rumbeiha W, Fitzgerald SD, Stein G, Baek SH, Goudreau J, Kassab M, Majid A (2012). Asiatic acid attenuates infarct volume, mitochondrial dysfunction, and matrix metalloproteinase-9 induction after focal cerebral ischemia. Stroke.

[22] Park BC, Bosire KO, Lee ES, Lee YS, Kim JA (2005). Asiatic acid induces apoptosis in SK-MEL-2 human melanoma cells. Cancer Lett.

[23] Wei J, Huang Q, Huang R, Chen Y, Lv S, Wei L, Liang C, Liang S, Zhuo L, Lin X (2013). Asiatic acid from Potentilla chinensis attenuate ethanol-induced hepatic injury via suppression of oxidative stress and Kupffer cell activation. Biol Pharm Bull.

[24] Gao J, Chen J, Tang X, Pan L, Fang F, Xu L, Zhao X, Xu Q (2006). Mechanism underlying mitochondrial protection of asiatic acid against hepatotoxicity in mice. J Pharm Pharmacol.

[25] Tang LX, He RH, Yang G, Tan JJ, Zhou L, Meng XM, Huang XR, Lan HY (2012). Asiatic acid inhibits liver fibrosis by blocking TGF-beta/Smad signaling *in vivo* and *in vitro*. PLoS One.

[26] Guo W, Liu W, Hong S, Liu H, Qian C, Shen Y, Wu X, Sun Y, Xu Q (2012). Mitochondria-dependent apoptosis of con A-activated T lymphocytes induced by asiatic acid for preventing murine fulminant hepatitis. PLoS One.

[27] Ma K, Zhang Y, Zhu D, Lou Y (2009). Protective effects of asiatic acid against D-galactosamine/lipopolysaccharide-induced hepatotoxicity in hepatocytes and kupffer cells co-cultured system via redox-regulated leukotriene C4 synthase expression pathway. Eur J Pharmacol.

[28] Yan SL, Yang HT, Lee YJ, Lin CC, Chang MH, Yin MC (2014). Asiatic acid ameliorates hepatic lipid accumulation and insulin resistance in mice consuming a high-fat diet. J Agric Food Chem.

[29] Guo W, Liu W, Jin B, Geng J, Li J, Ding H, Wu X, Xu Q, Sun Y, Gao J (2015). Asiatic acid ameliorates dextran sulfate sodium-induced murine experimental colitis via suppressing mitochondria-mediated NLRP3 inflammasome activation. Int Immunopharmacol.

[30] Bian D, Zhang J, Wu X, Dou Y, Yang Y, Tan Q, Xia Y, Gong Z, Dai Y (2013). Asiatic acid isolated from Centella asiatica inhibits TGF-beta1-induced collagen expression in human keloid fibroblasts via PPAR-gamma activation. Int J Biol Sci.

[31] Croasdell A, Duffney PF, Kim N, Lacy SH, Sime PJ, Phipps RP (2015). PPARgamma and the innate immune system mediate the resolution of inflammation. PPAR Res.

[32] Abdelrahman M, Sivarajah A, Thiemermann C (2005). Beneficial effects of PPAR-gamma ligands in ischemia-reperfusion injury, inflammation and shock. Cardiovasc Res.

[33] Hong W, Hu S, Zou J, Xiao J, Zhang X, Fu C, Feng X, Ye Z (2015). Peroxisome proliferator-activated receptor gamma prevents the production of NOD-like receptor family, pyrin domain containing 3 inflammasome and interleukin 1beta in HK-2 renal tubular epithelial cells stimulated by monosodium urate crystals. Mole Med Rep.

[34] Miao H, Ou J, Ma Y, Guo F, Yang Z, Wiggins M, Liu C, Song W, Han X, Wang M, Cao Q, Chung BH, Yang D (2014). Macrophage CGI-58 deficiency activates ROS-inflammasome pathway to promote insulin resistance in mice. Cell Rep.

[35] Boaru SG, Borkham-Kamphorst E, Tihaa L, Haas U, Weiskirchen R (2012). Expression analysis of inflammasomes in experimental models of inflammatory and fibrotic liver disease. J Inflamm (Lond).

[36] Kinoshita M, Uchida T, Sato A, Nakashima M, Nakashima H, Shono S, Habu Y, Miyazaki H, Hiroi S, Seki S (2010). Characterization of two F4/80-positive Kupffer cell subsets by their function and phenotype in mice. J Hepatol.

[37] Abais JM, Xia M, Zhang Y, Boini KM, Li PL (2015). Redox regulation of NLRP3 inflammasomes: ROS as trigger or effector?. Antioxid Redox Signal.

[38] Tschopp J (2011). Mitochondria: sovereign of inflammation?. Eur J Immunol.

[39] Hara H, Tsuchiya K, Kawamura I, Fang R, Hernandez-Cuellar E, Shen Y, Mizuguchi J, Schweighoffer E, Tybulewicz V, Mitsuyama M (2013). Phosphorylation of the adaptor ASC acts as a molecular switch that controls the formation of speck-like aggregates and inflammasome activity. Nat Immunol.

[40] Rajamaki K, Mayranpaa MI, Risco A, Tuimala J, Nurmi K, Cuenda A, Eklund KK, Oorni K, Kovanen PT (2016). p38delta MAPK: a novel regulator of NLRP3 inflammasome activation with increased expression in coronary atherogenesis. Arterioscler Thromb Vasc Biol.

[41] Hamada E, Nishida T, Uchiyama Y, Nakamura J, Isahara K, Kazuo H, Huang TP, Momoi T, Ito T, Matsuda H (1999). Activation of Kupffer cells and caspase-3 involved in rat hepatocyte apoptosis induced by endotoxin. J Hepatol.

[42] Huang X, Zuo L, Lv Y, Chen C, Yang Y, Xin H, Li Y, Qian Y (2016). Asiatic acid attenuates myocardial ischemia/reperfusion injury via Akt/GSK-3beta/HIF-1alpha signaling in rat H9c2 cardiomyocytes. Molecules.

[43] Peralta C, Jimenez-Castro MB, Gracia-Sancho J (2013). Hepatic ischemia and reperfusion injury: effects on the liver sinusoidal milieu. J Hepatol.

[44] Chang YP, Ka SM, Hsu WH, Chen A, Chao LK, Lin CC, Hsieh CC, Chen MC, Chiu HW, Ho CL, Chiu YC, Liu ML, Hua KF (2015). Resveratrol inhibits NLRP3 inflammasome activation by preserving mitochondrial integrity and augmenting autophagy. J Cell Physiol.

[45] Deng ZY, Hu MM, Xin YF, Gang C (2015). Resveratrol alleviates vascular inflammatory injury by inhibiting inflammasome activation in rats with hypercholesterolemia and vitamin D2 treatment. Inflamm Res.

[46] Akahori T, Sho M, Hamada K, Suzaki Y, Kuzumoto Y, Nomi T, Nakamura S, Enomoto K, Kanehiro H, Nakajima Y (2007). Importance of peroxisome proliferator-activated receptor-gamma in hepatic ischemia/reperfusion injury in mice. J Hepatol.

[47] Kuboki S, Shin T, Huber N, Eismann T, Galloway E, Schuster R, Blanchard J, Zingarelli B, Lentsch AB (2008). Peroxisome proliferator-activated receptor-gamma protects against hepatic ischemia/reperfusion injury in mice. Hepatology.

[48] Miglio G, Rosa AC, Rattazzi L, Collino M, Lombardi G, Fantozzi R (2009). PPARgamma stimulation promotes mitochondrial biogenesis and prevents glucose deprivation-induced neuronal cell loss. Neurochem Int.

[49] Miglio G, Rosa AC, Rattazzi L, Grange C, Camussi G, Fantozzi R (2012). Protective effects of peroxisome proliferator-activated receptor agonists on human podocytes: proposed mechanisms of action. Br J Pharmacol.

[50] Chuang YC, Lin TK, Huang HY, Chang WN, Liou CW, Chen SD, Chang AY, Chan SH (2012). Peroxisome proliferator-activated receptors gamma/mitochondrial uncoupling protein 2 signaling protects against seizure-induced neuronal cell death in the hippocampus following experimental status epilepticus. J Neuroinflammation.

[51] Massip-Salcedo M, Casillas-Ramirez A, Franco-Gou R, Bartrons R, Ben Mosbah I, Serafin A, Rosello-Catafau J, Peralta C (2006). Heat shock proteins and mitogen-activated protein kinases in steatotic livers undergoing ischemia-reperfusion: some answers. Am J Pathol.

[52] Kobayashi M, Takeyoshi I, Yoshinari D, Matsumoto K, Morishita Y (2002). P38 mitogen-activated protein kinase inhibition attenuates ischemia-reperfusion injury of the rat liver. Surgery.

[53] Yoshinari D, Takeyoshi I, Kobayashi M, Koyama T, Iijima K, Ohwada S, Matsumoto K, Morishita Y (2001). Effects of a p38 mitogen-activated protein kinase inhibitor as an additive to university of wisconsin solution on reperfusion injury in liver transplantation. Transplantation.

[54] Jiang W, Li M, He F, Yao W, Bian Z, Wang X, Zhu L (2016). Protective effects of asiatic acid against spinal cord injury-induced acute lung injury in rats. Inflammation.

[55] Ma ZG, Dai J, Wei WY, Zhang WB, Xu SC, Liao HH, Yang Z, Tang QZ (2016). Asiatic acid protects against cardiac hypertrophy through activating AMPKalpha signalling pathway. Int J Biol Sci.

[56] Wu K, Hu M, Chen Z, Xiang F, Chen G, Yan W, Peng Q, Chen X (2017). Asiatic acid enhances survival of human AC16 cardiomyocytes under hypoxia by upregulating miR-1290. IUBMB Life.

[57] A IJ, Jeannin E, Wahli W, Desvergne B (1997). Polarity and specific sequence requirements of peroxisome proliferator-activated receptor (PPAR)/retinoid X receptor heterodimer binding to DNA. A functional analysis of the malic enzyme gene PPAR response element. J Biol Chem.

[58] Ricote M, Li AC, Willson TM, Kelly CJ, Glass CK (1998). The peroxisome proliferator-activated receptor-gamma is a negative regulator of macrophage activation. Nature.

[59] Marx N, Schonbeck U, Lazar MA, Libby P, Plutzky J (1998). Peroxisome proliferator-activated receptor gamma activators inhibit gene expression and migration in human vascular smooth muscle cells. Circ Res.

[60] Hou Y, Moreau F, Chadee K (2012). PPARgamma is an E3 ligase that induces the degradation of NFkappaB/p65. Nat Commun.

[61] Abe Y, Hines IN, Zibari G, Pavlick K, Gray L, Kitagawa Y, Grisham MB (2009). Mouse model of liver ischemia and reperfusion injury: method for studying reactive oxygen and nitrogen metabolites *in vivo*. Free Radic Biol Med.

[62] Suzuki S, Toledo-Pereyra LH, Rodriguez FJ, Cejalvo D (1993). Neutrophil infiltration as an important factor in liver ischemia and reperfusion injury. Modulating effects of FK506 and cyclosporine. Transplantation.

[63] Kakarla SK, Fannin JC, Keshavarzian S, Katta A, Paturi S, Nalabotu SK, Wu M, Rice KM, Manzoor K, Walker EM, Blough ER (2010). Chronic acetaminophen attenuates age-associated increases in cardiac ROS and apoptosis in the Fischer Brown Norway rat. Basic Res Cardiol.

[64] Wang C, Blough ER, Arvapalli R, Dai X, Paturi S, Manne N, Addagarla H, Triest WE, Olajide O, Wu M (2013). Metabolic syndrome-induced tubulointerstitial injury: role of oxidative stress and preventive effects of acetaminophen. Free Rad Biol Med.

