# Supplementary material for: Asiatic acid protects against hepatic ischemia/reperfusion injury by inactivation of Kupffer cells via PPARγ/NLRP3 inflammasome signaling pathway
Source: Oncotarget. 2017 Sep 21;8(49):86339–55. doi: 10.18632/oncotarget.21151 (PMC5689689; doi:10.18632/oncotarget.21151)
Supplement: Supplementary file 1 [file oncotarget-08-86339-s001.pdf]

Asiatic acid protects against hepatic ischemia/reperfusion injury by inactivation of Kupffer cells via PPARγ/NLRP3 inflammasome signaling pathway

SUPPLEMENTARY MATERIALS

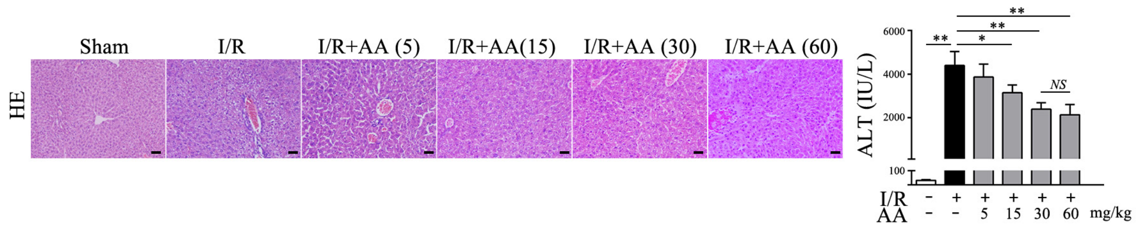

Supplementary Figure 1: Preliminary study for the optimal dose of AA on hepatic I/R injury. Male C57BL/6 mice were pretreated with either AA (5, 15, 30 and 60 mg/kg) or vehicle 1 h before hepatic I/R surgery. Representative histological staining of ischemic liver tissue and sALT were shown. Scale bar: 30μm. Results representative of 2-3 mice/group. *, ** Significant difference ($P < 0.05$, $P < 0.01$) compared between the indicated groups. NS No significant difference ($P > 0.05$) between the indicated groups.
